# Nest-associated scent marks help bumblebees localizing their nest in visually ambiguous situations

**DOI:** 10.3389/fnbeh.2023.1155223

**Published:** 2023-06-14

**Authors:** Sonja Eckel, Martin Egelhaaf, Charlotte Doussot

**Affiliations:** Department of Neurobiology, Faculty of Biology, Bielefeld University, Bielefeld, Germany

**Keywords:** navigation, homing, multisensory integration, vision, olfaction, insect

## Abstract

Social insects such as ants and bees are excellent navigators. To manage their daily routines bumblebees, as an example, must learn multiple locations in their environment, like flower patches and their nest. While navigating from one location to another, they mainly rely on vision. Although the environment in which bumblebees live, be it a meadow or a garden, is visually stable overall, it may be prone to changes such as moving shadows or the displacement of an object in the scenery. Therefore, bees might not solely rely on visual cues, but use additional sources of information, forming a multimodal guidance system to ensure their return home to their nest. Here we show that the home-finding behavior of bumblebees, when confronted with a visually ambiguous scenario, is strongly influenced by natural scent marks they deposit at the inconspicuous nest hole when leaving their nest. Bumblebees search for a longer time and target their search with precision at potential nest locations that are visually familiar, if also marked with their natural scent. This finding sheds light on the crucial role of odor in helping bees find their way back to their inconspicuous nest.

## 1. Introduction

The ability to find back home after foraging, often referred to as homing, is crucial for many animal species. The complexity of natural environments can make it a challenging task, being confronted with a large variety of stimuli from different modalities. To solve this task, a considerable number of navigational strategies evolved in the animal kingdom. The capabilities of central place foragers like ants, bees, and wasps are particularly interesting. Unlike many other insects, central place foragers live in nests, to which they need to return after foraging. This makes them good study organisms for navigation. Despite their relatively small brains (Witthoeft, 1967), they are excellent navigators and can learn multiple locations and routes of up to several kilometers length in their environment (Beekman and Ratnieks, 2000; Wikelski et al., 2010).

For diurnal foragers, the visual scenery around the goal location plays the major role for spatial navigation (Zeil, 2012; Chakravarthi et al., 2016). However, small objects, which the insects learned along their route, might be displaced by wind or other animals during their absence, making parts of the visual environment unstable. In such a situation, visual homing may become less reliable (Narendra and Ramirez-Esquivel, 2017), enforcing the foragers to back up on other cues to navigate back to the goal location. This becomes specifically crucial when it comes to the detection of the nest entrance. As an example, buff-tailed bumblebees, *Bombus terrestris*, usually live underground in small nests, mostly in former mouse holes. The entrance to such a nest is inconspicuous due to its small size and often hidden under grass or leaves (Foster and Gamboa, 1989). Therefore, in most cases the entrance is not visible to the bees. If the learned visual environment of the nest entrance changes during the absence of a forager, returning home would be considerably more difficult or possibly unfeasible, when relying solely on visual information.

However, the natural environment of bumblebee nests is not exclusively determined by visual cues, but may also be distinguished by information from other sensory modalities. Olfactory cues have been shown to play a decisive role for goal-finding behavior in many ecological contexts. For instance, ants associate environmental odors with important locations, like their nest entrance or a route they traveled (Steck et al., 2009; Buehlmann et al., 2013, 2015; Huber and Knaden, 2017). Integrating visual and olfactory information can facilitate learning of a place and enable a return even if one of the learned cues is missing (Steck et al., 2011). The integration of multimodal information is important not only when a cue disappears, but as well when it changes and therefore becomes less reliable. As an example, Huber and Knaden (2017) showed that ants weight the informative value of cues by their reliability and focus on the less ambiguous one to successfully return to a known location. However, not only ants, but also bees, including bumblebees, enhance their learning performance and decision making by integrating odor and visual cues during food detection (Chaffiol et al., 2005; Kulahci et al., 2008) and nest recognition in dense nesting regions (Foster and Gamboa, 1989; Ostwald et al., 2019; Frahnert and Seidelmann, 2021). In the latter context, both active and passive marking of the nest entrance and its immediate surrounding area play a key role, a behavior called home-range marking (Butler et al., 1969; Jandt et al., 2005; Guédot et al., 2006; Steck, 2012). For example, solitary bees place specific mixtures of their own scent as well as scents from the environment, e.g., from flowers visited before, inside the nest entrance to distinguish their nest from others when entering it (Rottler et al., 2013; Frahnert and Seidelmann, 2021). Bumblebees were also found to leave scent traces at the nest entrance while passing it. As an example, Foster and Gamboa (1989) tested whether bumblebees distinguish their own scent marks from scent marks of another bumblebees’ nest, each placed at one of two visually identical false nest tubes. Bumblebees were found to prefer the nest tube with the scent from their own nest, showing that they were able to distinguish their own scent from that of other colonies. The scent marks found at a bumblebee’s nest entrance consist of a composition of fatty acids, cuticular hydrocarbons and other chemicals, which are deposited by glands all over the body of the animals and mainly serve as protection from dehydration and infections (Hefetz et al., 1996; Goulson et al., 2000). Nevertheless, bumblebees were shown to learn to attribute different meanings to these scent marks in association with other cues and show adaptive behavior at multiple locations with the same chemicals (Saleh et al., 2007). For instance, the scent marks usually found at the nest entrance may also be used to mark food sources during foraging (Schmitt and Bertsch, 1990; Schmitt et al., 1991), or to avoid depleted flowers (Saleh et al., 2007). These chemicals are most likely perceived via contact chemoreception with the antennae (Foster and Gamboa, 1989). Several studies showed that walking bees use these scent marks for route following in specific contexts, for example, to find back the core of the nest from the entrance (Cederberg, 1977) or to travel between a food source and the nest even in the absence of visual landmarks (Chittka et al., 1999).

Despite the evidence for the role of olfactory cues in goal-finding, it is not known if bumblebees use scent marks deposited around their nest to localize its entrance during the final homing phase. When visual cues are available, bumblebees strongly rely on them for navigation, and studies on local homing in bees have so far mainly focused on the role of the visual scenery (Cartwright and Collett, 1983; Wehner, 2008; Hempel de Ibarra et al., 2009; Collett et al., 2013; Zeil, 2022). However, visual cues may change over time, e.g., if they are displaced by a gust of wind, and then lose their reliability. When bumblebees were trained to find their nest hole in a particular environment and then the different visual elements were placed in conflict to each other, they searched for their nest in several locations. These search locations corresponded to the area in which the nest entrance was learned to be relative to each of the visual cues (Doussot et al., 2020). Hence, a change in the learned visual scenery leads to more than one possible nest location. In such visually ambiguous situations the bees will have a lower chance to find the inconspicuous nest entrance. However, if the nest entrance was characterized by other than visual information, the bumblebees might have the option to focus on another sensory modality and potentially return back home successfully.

We analyzed whether scent marks, deposited by bumblebees around the nest entrance, affect their homing behavior in a visually ambiguous environment, using the same setup as Doussot et al. (2020). We hypothesized that bumblebees associate their nest entrance with the scent marks and use these scents to locate the nest when the learned visual cues are not reliable. Our analysis confirms that when the visual cues were brought into conflict during the test, bumblebees searched for a longer time at the scent-marked potential nest location and were more precise in targeting the potential nest if marked by the scent.

## 2. Results

**FIGURE 1:**
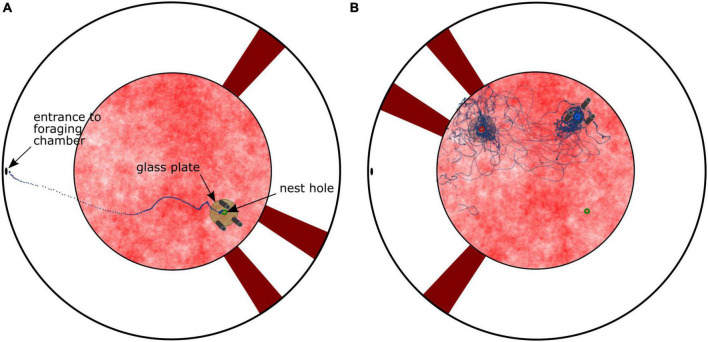
Top view of the cylindrical arena showing the two sets of cue constellations (cylinders vs. stripes on the arena wall). **(A)** Training condition. The dotted line represents an example trajectory of a homing bumblebee flying from the entrance hole of the foraging chamber to the nest (green). **(B)** Test condition with visual conflict. The cylinder constellation was rotated by 90°counterclockwise and the stripe constellation by 180°. The blue circle indicates the position at which the nest was learnt relative to the cylinder constellation (cylinders-associated nest hole), the red circle relative to the stripe constellation (stripes-associated nest hole), respectively. The real nest entrance (green) was covered with the same pattern as the floor. The dotted line represents an example trajectory of a single bumblebee entering the arena from the foraging chamber. The bee searched at both fictive nest holes alternately. The real nest entrance was not approached.

**FIGURE 2:**
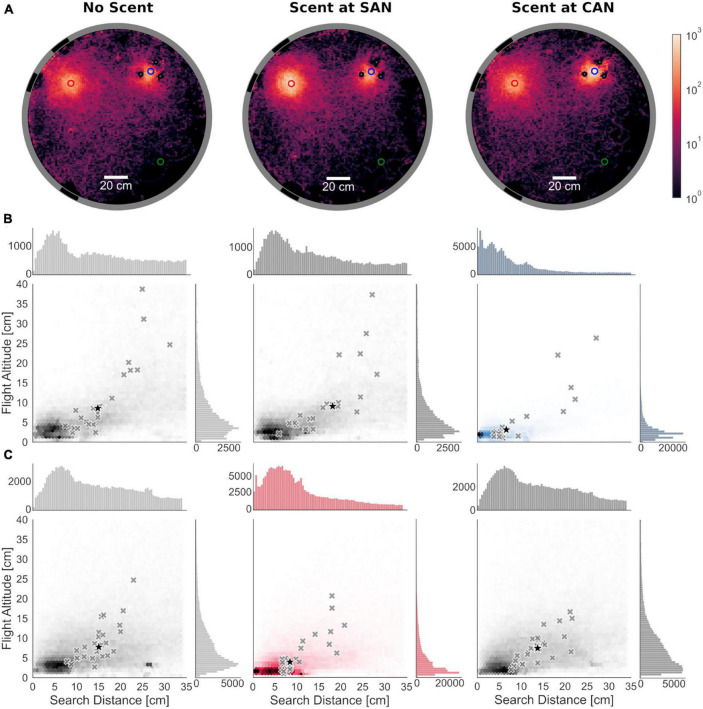
Search distribution around landmark-associated nest entrance locations pooled from all animals (*N* = 25). **(A)** Top view of the arena showing the heatmaps of the spatial distribution of all trajectories in the x-y-plane for the different test conditions (indicated above the heatmaps); the number of stays of the bees at each point of the hexagonal grid that spanned the horizontal plane was counted and plotted along a logarithmic scale for visualization purposes. The scale is represented by a color gradient that ranges from black, the minimum, to bright red, the maximum. When scent was placed at either the cylinders-associated (CAN) or the stripes-associated fictive nest entrance (SAN), bumblebees focused their search on the scent-marked one. Without scent marks the search was more widely distributed around both fictive nest entrances with the bees searching more at the SAN than at the CAN. **(B)** Search distributions at CAN: Search density of all bumblebees along the distance to the fictive nest entrances in the horizontal plane (*x*-axis) and flight altitude (*y*-axis) in a range of 34 cm around the cylinders-associated nest hole. Gray cross markings show the medians of search distributions of individual bees. The black star indicates the median across all tested bees of the medians of the search distributions. Histograms above and right to the main plots show the distribution of all data points of the search distance (top) and flight altitude (right). The search distributions are shown in blue if the CAN was marked with scent. **(C)** Search distributions at SAN. The data are shown in the same format as in panel **(B)**. The search distributions are shown in red if the SAN was marked with scent. **(B,C)** The search distance and flight altitude at both fictive nest locations were smaller with scent marks than without.

We trained bumblebees indoors to travel from their nest entrance in the ground of a cylindrical flight arena to a foraging chamber connected to a hole in the upper margin of the arena via a tube system. The arena contained two different visual landmark constellations to allow the bee to pinpoint the inconspicuous nest entrance. One constellation consisted of three cylinders (15 cm height, 2.5 cm diameter), which were arranged around the nest entrance at 10 cm distance and served the bees as local landmarks. The second landmark constellation consisted of three vertical stripes (85 cm height, 12 cm width), which were fixed to the white arena wall and were used as more distant landmarks. After some days bees normally flew straight back to their nest entrance when returning from foraging (Figure 1A). After we relocated the two landmark constellations relative to each other and away from the nest hole, hence generating a visual conflict, the bees searched, in accordance with Doussot et al. (2020), alternately at two locations in the arena, as is shown for an individual flight in Figure 1B and for the entire population of tested bees by the search distribution in Figure 2A (left diagram). These search locations correspond to the position where the nest hole used to be relative to each landmark constellation during training. Therefore, we refer to these locations as landmark-associated fictive nest holes. The real nest entrance was covered with the same pattern as the arena floor; thus, it was no longer visible. Consequently, the real nest was never approached during the tests. In this kind of paradigm, the bumblebees likely located their nest based on the visual landmark constellations as described above, rather than using potential compass cues, since most external structures in the lab were covered by white fabric.

We wanted to test whether bumblebees use self-laid scent marks in combination with visual information to locate their nest in this visually ambiguous scenario. Consequently, we placed a glass plate around the nest entrance to collect scent marks that were passively laid by bumblebees while walking on it after leaving the nest (see also Saleh et al., 2007). Each bee was tested under three conditions. During two of the test conditions, we placed the scent-marked plate at either of the fictive nest holes. The other landmark-associated nest hole was surrounded by a visually identical clean glass plate. In the third condition, which served as a control, we placed visually identical clean plates at both fictive nest holes. To ensure that the glass plate contained scent marks, we analyzed a sample collected after 3 days via gas chromatography/mass spectrometry (GC/MS). We found a large variety of cuticular hydrocarbons, fatty acids, and further substances that were already discovered in other studies on the composition of bumblebee scent marks (Hefetz et al., 1996; Goulson et al., 2000).

We scrutinized two characteristics of the bumblebees’ flight trajectories in the region surrounding the two fictive nest locations: (a) the spatial search distribution and (b) the search duration within a radius of 10 cm around each fictive nest hole.

### 2.1. Do scent marks alter the spatial search distribution of bumblebees?

In the absence of scent marks, bumblebees searched for their nest at the two fictive nest holes. If the scent marks were placed at either of the nest holes, the bees focused their search on the scent-marked region (Figure 2A). To investigate the difference of search patterns between scent-marked and scent-free fictive nests in more detail, we analyzed the search distance not only in the horizontal plane but also the flight altitude around the fictive nests. To assign each point of the trajectory to only one of the fictive nest holes, we limited the analysis to an area within a radius of 34 cm around each fictive nest hole, corresponding to half of the distance between both locations.

The search distributions over the entire flights of all animals indicate that when the animals search close to the fictive nest entrance in the plane, they do so at a low flight altitude. This impression is confirmed by the position of the medians of the search distributions of the individual bumblebees (Figures 2B, C). Here, too, the flight altitude increases with increasing distance from the fictive nest entrance (Spearman’s rank correlation based on the medians of search distributions of the individual bees, *r*-values range from 0.73 to 0.91, *p* < 0.001 for all fictive nests and conditions). This general conclusion holds regardless of whether the experiments were conducted under visual cue conflict conditions alone, or whether scent was presented at one of the nest entrances.

The scent marks had a clear effect on how focused the spatial distribution of the bumblebees’ search behavior at the two fictive nest entrances were. For each fictive nest location, we made pairwise comparisons of the median search distance and altitude for the search flights of each animal between conditions, in which no scent was present at any of the fictive nest entrances (control condition) and in which the scent was placed at the corresponding fictive nest entrance, respectively. At both landmark-associated nest holes, the median search distance to the corresponding nest hole significantly decreased when scent marks were present (two-sided Wilcoxon signed-rank test; cylinders-associated nest: *z* = −2.78, *p* = 0.004; stripes-associated nest: *z* = −3.54, *p* < 0.001), showing a strong shift toward the center of the scent-marked region, i.e., the fictive nest hole. At the cylinders-associated nest hole, the median distance of the pooled data of all animals decreased from 14.32 cm without scent to 5.76 cm with scent marks (based on pooled data shown in Figure 2C). At the stripes-associated nest hole a decrease from 13.58 to 8.62 cm could be observed (Figure 2B). Like the search distance in the horizontal plane, the median flight altitude significantly decreased at both fictive nest holes when they were scent-marked (two-sided Wilcoxon signed-rank test; cylinders-associated nest: *z* = −2.25, *p* = 0.02, stripes-associated nest: *z* = −2.68, *p* = 0.006). The median flight altitude of pooled data from all animals decreased from 6.86 cm without scent to 2.53 cm with scent at the cylinders-associated nest (Figure 2C) and from 6.75 to 3.50 cm at the stripes-associated nest (Figure 2B).

### 2.2. Do scent marks alter the search duration of bumblebees?

**FIGURE 3:**
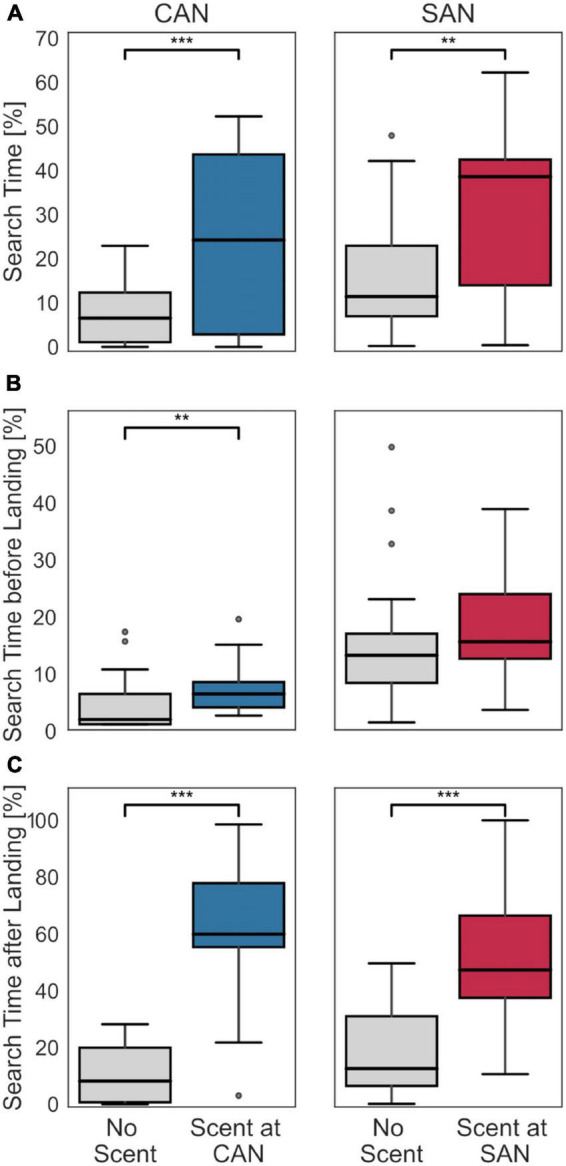
Search time at the fictive nest locations with and without scent. **(A)** Percentage of search time at the fictive nest entrances (CAN left panel, SAN right panel) during the entire return flight under the condition without scent marks at any fictive nest entrance (left diagram in each panel) and with scent marks at the respective fictive nest entrance (right diagram in each panel). The search time significantly increased at both fictive nest entrances when scent marks were placed at the corresponding position. **(B)** Percentage of search time before the first landing under the control condition without scent marks at any of the fictive nest entrances (left diagram in each panel) and at the respective fictive nest entrance with scent marks (right diagram in each panel). The search duration at the cylinders-associated nest slightly increased when scent was present. At the stripes-associated nest no significant difference could be found. **(C)** Percentage of search time after the first landing. The data are shown in the same format as in panel **(B)**. The overall search time after landing is significantly higher when scent is present. In case of statistically significant differences between the two distributions, the respective significance level is indicated at the top of the subfigure: *** indicating *p* < 0.001 and ** indicating *p* < 0.01.

To assess whether scent marks also affect the time bees search close to the two fictive nest holes, we determined the proportion of the flight that bees spent searching for their nest at each location. Since we wanted to investigate the homing behavior of bumblebees, rather than flights in the arena not related to the nest, we concentrated our analysis on the flights below an altitude threshold of 17.13 cm [i.e., 75% of the data distribution along the altitude, as in Doussot et al. (2020); see Supplementary Figure 1]. In this way we could exclude potential unrelated behavior such as escape behavior and collisions with the arena lid. For each fictive nest hole, we made pairwise comparisons for all bees between search times in the vicinity of the nest holes under the control condition without scent marks at any nest entrance and the conditions with scent marks at one of the nest entrances, respectively. The region for analyzing the search at a fictive nest hole was set to a radius of 10 cm around each fictive nest entrance in the horizontal plane, corresponding to the size of the glass plate used for scent collection (larger radii were additionally tested and did not show qualitatively different results; data not shown). Equivalent to the search density, the presence of scent increases the search time of bumblebees in the scent-marked region (Figure 3A). For both fictive nest holes there is a more than threefold increase in the median search time between the scent-free and scent-marked nest hole [Wilcoxon signed-rank test; scent-free (6.5%) versus scent-marked cylinders-associated nest (24.23%): *z* = −3.53, *p* < 0.001, scent-free (11.41%) versus scent-marked stripes-associated nest (38.63%): *z* = −2.84, *p* = 0.002].

### 2.3. Which role does physical contact play for scent detection?

The previous results already indicated that the olfactory information mainly comes into play when the bees fly close to the odor source. However, it remains unclear when exactly bumblebees detect the scent marks. Given that these scent marks mainly consist of long-chained, poorly volatile hydrocarbons (Hefetz et al., 1996; Goulson et al., 2000) and most of the bees landed on the scent-marked glass plate (18/25 at scented CAN, 20/25 at scented SAN), we hypothesize that physical contact with it, such as with their antennae, may play a role in identifying the scent.

If this were the case, we might expect changes in the search behavior at the fictive nest locations after the bees landed on the scent-marked plate in comparison to when no scent was present. In contrast, before the bees landed the search behavior should be very similar. As a behavioral indicator for such a change, we compared the time spent searching at each fictive nest location before and after the first landing between the control condition without scent marks at any nest entrance and the condition with scent marks at the corresponding nest entrance, respectively, (Figures 3B, C). The time before landing refers to the total duration of all flight segments below the defined altitude threshold (see the section “2.2. Do scent marks alter the search duration of bumblebees?”) until the first landing. The time after landing refers to the flight duration from the landing until the end of the flight. Since recording times were limited to 5 min, and bees first landed at different time points in each recording, the search durations were normalized to the corresponding flight duration for each recording. The results of our comparisons show that the relative search time before landing (Figure 3B) differs only slightly with and without scent marks for both fictive nests: it increased by 4.5% for the cylinders-associated nest (Mann–Whitney U-test; *z* = 2.58, *p* = 0.01) and by 2.4% for the stripes-associated nest (Mann–Whitney U-test; *z* = 1.66, *p* = 0.1). In contrast, at both fictive nest locations the relative search times after landing increased considerably more in the presence of scent than in the control, i.e., by 51.8% for the cylinders-associated nest (Mann–Whitney U-test; *z* = 4.78, *p* < 0.001) and by 34.8% for the stripes-associated nest (Mann–Whitney U-test; *z* = 3.75, *p* < 0.001).

**FIGURE 4:**
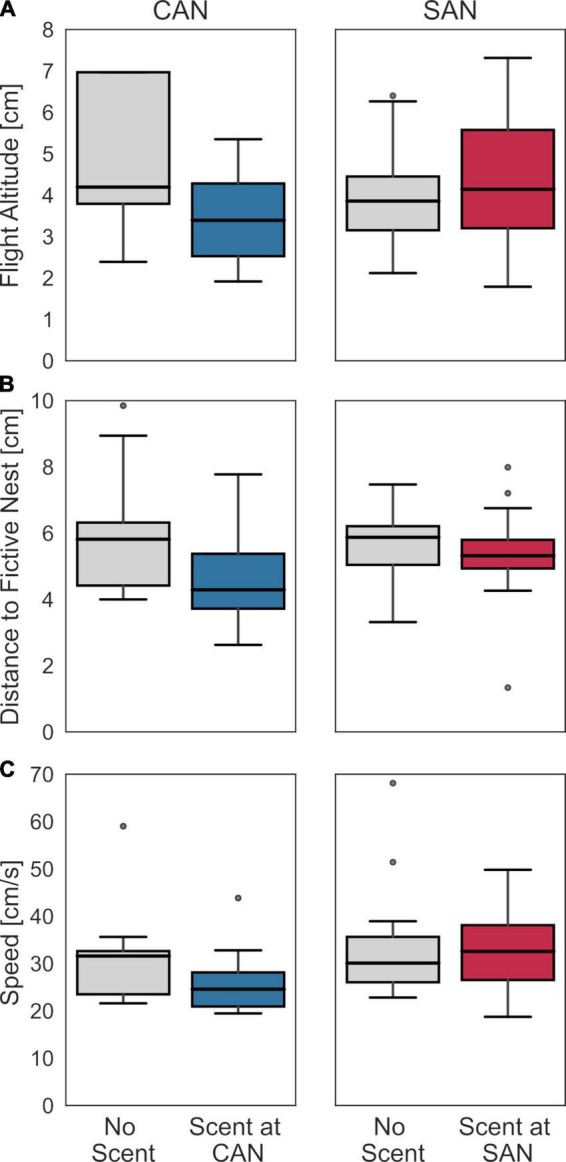
Search behavior before landing with and without scent at the fictive nest entrance. **(A)** Flight altitude, **(B)** flight distance to the fictive nest hole in the horizontal plane, and **(C)** flight speed during the last 5 s of flight before landing for the condition without scented fictive nest entrance (left diagrams in each panel) and for the condition with either scent at CAN (left panel) or at SAN (right panel), respectively, (right diagrams in each panel). The search behavior did not differ systematically between the control condition without scent marks and the corresponding condition with scent.

To further corroborate the hypothesis that landing on the scent-marked plate is primarily responsible for the observed change in search behavior, we examined how similar the overall flight behavior prior to landing was in the control condition and the respective condition with scent. This comparison was performed for three indicators: the distance to the fictive nest entrance in the horizontal plane, the flight altitude, and the flight speed for a time-window of 5 s before landing (Figure 4). No systematic and significant differences were found for any of these behavioral characteristics (Mann–Whitney U-test; landing at CAN: altitude: *z* = −0.32, *p* = 0.76; distance: *z* = −0.61, *p* = 0.55; speed: *z* = −1.41, *p* = 0.16; landing at SAN: altitude: *z* = 0.58, *p* = 0.6; distance: *z* = −0.12, *p* = 0.95; speed: *z* = 0.34, *p* = 0.77). To ensure that the result is not dependent on the selected time window, we did the same analysis for time windows of 1 s and 10 s, respectively. Apart from one exception, where the flight speed in the final second before landing at the cylinders-associated nest was found to decrease slightly (from 25 to 20 cm/s) but significantly when scent was present (*z* = −2.17, *p* = 0.03), there were no significant dependences between the behavioral indicators and the presence or absence of scent at a fictive nest location. Notwithstanding, these findings are generally consistent with our hypothesis that the scent might only be identified during flight in immediate ground proximity or by direct physical contact after the bee has landed on the scent-marked plate.

## 3. Discussion

In the light of previous findings showing that bumblebees deposit scent marks at their nest entrance when leaving for a foraging trip (Rollo et al., 1995; Ginzel et al., 2006; Aksenov and David Rollo, 2017; Funaro et al., 2018), we wanted to explore whether and how such scent marks influence the strongly visually driven homing behavior of bumblebees. It is known that in many bee species self-laid scent marks are important for the final recognition of their own nest hole after entering it (Foster and Gamboa, 1989; Rottler et al., 2013). Here, we asked if the role of scent marks extends to an earlier stage of homing behavior; we wanted to explore whether these odor cues play a role in helping bees to locate the entrance of their nest, which is a critical first step for them to return to their home. Since visual cues play a well-established key role in finding the nest hole (see the section “1. Introduction” and below), we investigated the question of multimodal integration in homing behavior by means of a cue conflict paradigm (Doussot et al., 2020). Here the visual information was no longer unambiguous allowing the odor information to potentially play the decisive role in resolving the visual ambiguity. In this way, we could show that in such a conflict situation, where the visual cues indicate two possible nest hole positions, self-laid scent marks lead the bumblebees to search preferentially at the scent-marked location. Thus, olfactory information largely resolves the visual ambiguity.

This novel finding has immediate consequences for our understanding of the mechanisms underlying homing behavior. Central-place foragers are generally assumed to learn panoramic snapshots of the visual scenery at or in the vicinity of important locations, like their nest or feeding sites, and use these snapshots for comparison with the currently perceived visual panorama to determine the home direction (Zeil, 2012). Many variants of this basic model have been used to simulate homing behavior of foraging insects (Baddeley et al., 2012; Kodzhabashev and Mangan, 2015; Le Möel and Wystrach, 2020). However, most of these vision-based models could not fully explain homing behavior under various environmental conditions. For example, it could be shown that in the cue conflict situation, as used in the present study, models based on a single memorized panoramic view of the nest surroundings could not account for the bumblebees’ search location. Although homing models relying on multiple views performed better, with fewer snapshots needed if optic flow-based spatial information was encoded and learned, rather than just brightness information, even in the absence of a visual conflict these models failed to lead the simulated bee to the location of the nest entrance (Doussot et al., 2020). This suggests that bees might use further information to pinpoint their visually inconspicuous nest. Altogether, in order to explain the finding of the current study that olfactory information helps to resolve the visual conflict, the output signals of the visual homing mechanism might be integrated with the olfactory input (Chandrasekaran, 2017).

Olfactory cues are relevant in the functional context of homing only in the very near range. In our findings bumblebees concentrated their search on a small volume around the scent-marked fictive nest location and may use it as local cue to pinpoint the nest hole. Because after landing at the scent-marked fictive nest the search time in the vicinity of the assumed nest entrance increased considerably compared to the situation without scent, one might conclude that physical contact supports the detection and identification of the scent. This conclusion is warranted by the fact that scent marks of bumblebees mostly contain poorly volatile substances (Foster and Gamboa, 1989; Frahnert and Seidelmann, 2021). However, the subtle differences in search behavior at the two fictive nest holes suggest that scent marks, despite their limited volatility, might already be partially detected while in flight near the ground. Although the search duration at the stripes-associated nest did not increase significantly in the presence of scent, there was a slight increase in search time at the cylinders-associated nest when scent was present (Figure 3B). Since bees flew slightly closer to the ground before landing at the cylinders-associated nest hole than at the stripes associated nest (Figure 4A), this may have facilitated the detection of scent components and led to a slight increase in search time at the cylinders-associated nest. Irrespective of these subtle details, it is safe to conclude on the basis of our results that visual information is used for approaching the approximate location of the nest from a greater distance, while the scent is used for pinpointing the nest entrance in the near range, i.e., after landing or already when flying close to the ground. This assumption is supported by previous studies on the use of self-laid scent marks in different functional contexts, which indicated that these might only be detected in a small volume or by physical contact (Cameron, 1981; Foster and Gamboa, 1989; Schmitt and Bertsch, 1990; Stout and Allen, 1998; Guédot et al., 2006; Frahnert and Seidelmann, 2021). It is likely that the antennae of social insects play a major role in detecting the colony-dependent scents that are deposited at the nest hole, but also on flowers to indicate that they have been depleted. Antennae have been shown to possess chemoreceptors, that are specialized to cuticular hydrocarbons as found in insect secretions (Pask et al., 2017).

Although our finding that olfactory cues are functionally relevant to spatially pinpoint the location of the bumblebee nest are novel, the combination of visual and olfactory information in other functional contexts appears to be well established for central place foragers, where olfactory cues open up options that are not visually possible. When it comes to finding food sources, animals can benefit greatly from using both visual and olfactory cues to navigate through their environment. In fact, research has shown that this combination of sensory information is particularly helpful when searching for flowers that are rich sources of nectar or pollen, due to the diverse array of scents that they emit (Buehlmann et al., 2013, 2020; Sprayberry, 2018; Ostwald et al., 2019). By using both their eyes and antennae bees can more easily locate the most profitable flowers and maximize their chances of obtaining the sustenance they need to survive. Combining multiple sensory modalities also gives animals the chance to use different cue components depending on their reliability. Cue reliability and its usage by central-place foragers was already addressed in several recent studies (Wolf and Wehner, 2005; Merkle et al., 2006; Huber and Knaden, 2017; Hoinville and Wehner, 2018). For example, insects were concluded to weight cue components by their informative value and have the possibility to revert to one of the cues, when the other one becomes less reliable or disappears (Huber and Knaden, 2017). This supports our conclusion that bumblebees assign a great weight to olfactory cues to guide them when the information brought by visual cues is uncertain or ambiguous. This highlights the importance of using multiple sources of sensory information to navigate complex environments.

## 4. Materials and methods

For the experiments, 25 individually marked animals from two healthy hives of *Bombus terrestris* provided by Biobest Group NV, Belgium, and Koppert B.V., Netherlands, were used. Each hive was kept in an acrylic box, covered with dark cloth to simulate the natural underground habitat of *B. terrestris*. The exit-tube of the nest box was connected to an opening in the plexiglass floor of a cylindrical arena of 150 cm diameter and 85 cm height. The arena’s ceiling was made of transparent plexiglass, through which we recorded the bees at 62.5 frames per second with four synchronized high-speed cameras (Basler acA 2040 um-NIR) arranged in different orientations around the setup to ensure that the entire arena was captured. From the recordings of the four calibrated cameras, we tracked the bee’s position in the video frame by using a custom-made Python script, based on OpenCV. The room was artificially illuminated with light tubes (Osram Natura L 58W/76) in a natural 12 h day-night cycle. Windows were covered with blinds to exclude additional sunlight. Around the upper edge of the arena wall, eight LEDs (GreenLED 5.5 W, 515 lm) were arranged to evenly illuminate the setup. Additionally, light tubes (Osram Natura T8 36W/76) were placed below the arena to simplify the tracking of the bees. These were covered with a red filter, so that the light could not be perceived by the bees (Skorupski and Chittka, 2010). On the plexiglass floor we placed a random red and white pattern [with a 1/f spatial frequency distribution (pink noise)] printed on backlight foil to let the light from the floor go through. The arena walls were entirely white. To avoid the bees using external visual cues, the area above the setup was covered with white fabric.

### 4.1. Training

The bees were trained to two sets of visual landmarks. One set consisted of three vertical stripes (85 cm height, 12 cm width), which were fixed to the white wall. They were made of red acrylic plastic ensuring the video tracking of a black target (the bee) against its background while providing the bees with strong contrast (Skorupski and Chittka, 2010). Two of these stripes were placed close to the nest entrance while the third was more distant to the nest (see Figure 1). The second set of visual cues consisted of three dark gray cylinders (15 cm height, 2.5 cm diameter), which were arranged around the nest entrance at 10 cm distance. Additionally, a glass plate (10 cm radius) with a 1 cm large hole in the center was placed directly around the nest entrance to collect scent marks of bumblebees leaving or entering the nest. This scent-covered disk was later used as odor cue for the tests.

From the cylindrical arena, the bees had access to a foraging chamber located at the plexiglass’s lid height, in the direction almost opposite to the nest. In the chamber they were supplied with pollen and 30% sucrose solution *ad libitum* via gravity feeders.

Before the test phase started, we let the bees habituate to the arena for 1 week. During that time, they could learn the location of their nest entrance in the visual scenery and lay scent marks on the glass plate. To ensure that the glass plate contained scent marks, we analyzed multiple samples via gas chromatography/mass spectrometry (data not shown). We found a large variety of fatty acids and cuticular hydrocarbons that were already known to be produced by bumblebees (Hefetz et al., 1996) and are partially assumed to be used as odor cues (Rollo et al., 1995; Ginzel et al., 2006; Aksenov and David Rollo, 2017; Funaro et al., 2018). We, therefore, presumed that the scent sample carried by the glass plate can be used as odor cue during experiments.

### 4.2. Test

We positioned the two sets of learned visual landmarks in a conflicting arrangement (Figure 1B) to test whether bumblebees use scent marks laid by conspecifics around the nest to find its entrance in visually ambiguous situations, i.e., the landmarks indicated more than one possible nest location. In this ambiguous situation, we recorded individual return flights of bumblebees. To do so, we released one bee at a time into the setup and recorded its return flight for 5 min. The position of the bee was tracked in each video frame with a custom-made Python script and manually corrected when needed.

Before these recordings were performed, we blocked bees inside the foraging chamber while feeding. We measured the time spent in the foraging chamber to ensure that there was no decrease in motivational state correlated with increased time spent (see Supplementary Figure 2). While the bees were blocked and could not perceive the setup, the arena was emptied and cleaned with 70% absolute ethanol solution to remove any additional odor cues on the arena floor. Afterward, the nest entrance was camouflaged with the floor pattern, and the cues were displaced at two different locations in the arena as in Figure 1B. For two test conditions, the glass plate containing the scent marks was placed at either of the two landmark-associated nest locations, i.e., the position of the nest relative to the visual cue, which the bees learned during the training phase. The other location was equipped with a clean plate to compensate for the potential effect of the glass plate as a visual or tactile cue. For the third condition, which was used as a control, we placed a clean glass plate at each of the landmark-associated nests to test the homing behavior of bees when no odor cue is accessible.

We tested each bee once per condition with at least 1 day break between tests to assure a renewed habituation to the trained cue constellation. For data analysis only those 25 of the 37 bees involved in the experiments were used that could be tested under all three conditions in a pseudo-randomized order to allow a pair-wise comparison of the behavior between conditions. The analysis of the effects of the first landing at the fictive nest locations on the search behavior (Figure 3B) and the analysis of flight characteristics of the immediate pre-landing phase (Figure 4) were performed only for bees that landed at the scented plate in the corresponding test condition, i.e., 20 bees for the condition scent at SAN, 18 bees for the condition scent at CAN and 22 bees for the control condition without scent. The evaluation and statistical analysis of the collected data was performed using Python (version 3.7.1).

## Data availability statement

The raw data supporting the conclusions of this article will be made available by the authors, without undue reservation.

## Author contributions

SE and CD designed the bumblebee experiments and built the experimental setup. SE conducted the experiments and data analysis supervised by CD. SE wrote the first draft of the manuscript. All authors conceptualized the project, contributed to the manuscript revision, read, and approved the submitted version.

## Supplementary material

Supplementary Figure 1 Overall flight distribution of all data along the *z*-axis. **(Left)** Data distribution along the *z*-axis and *x*-axis of the arena. The red line represents the limit below which the bees spent 75% of the time of all recordings. Below the limit, the flight of the bee is considered as search behavior. Data points above the red line can be mainly explained by random flights or bees trying to escape and colliding with the transparent roof of the arena. **(Right)** Histogram of the flight probability along the *z*-axis of the arena. The data show that bees were flying close to the floor for most of the time, with 75% of the data laying below 17.13 cm.

Supplementary Figure 2 Effect of the time the bees were blocked before their return flight on the duration of searching. The time spent in the foraging chamber has no significant effect on the search behavior, i.e., the time of the recording that was spent below 17.13 cm (linear regression; slope: 0.06, *p* > 0.1).
