# Supplementary figures and images for: Nest-associated scent marks help bumblebees localizing their nest in visually ambiguous situations
Source: Front Behav Neurosci. 2023 Jun 14;17:1155223. doi: 10.3389/fnbeh.2023.1155223 (PMC10300278; doi:10.3389/fnbeh.2023.1155223)

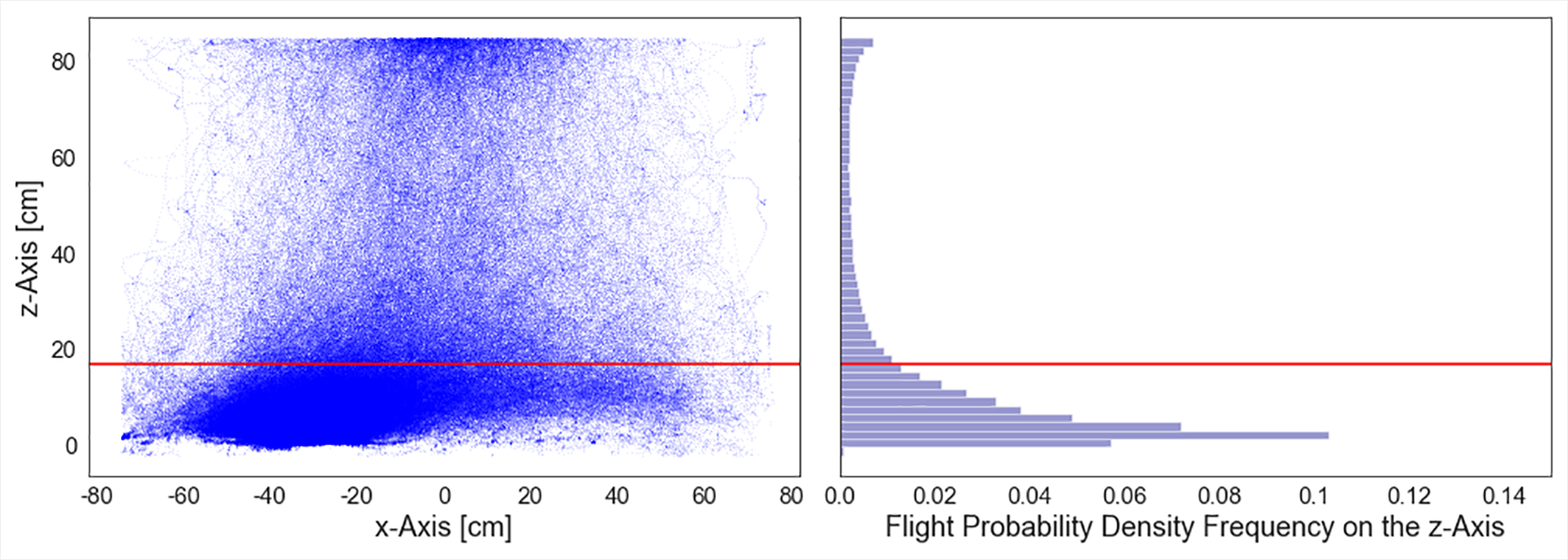

Supplement: Supplementary Figure 1 — Overall flight distribution of all data along the z-axis. (Left) Data distribution along the z-axis and x-axis of the arena. The red line represents the limit below which the bees spent 75% of the time of all recordings. Below the limit, the flight of the bee is considered as search behavior. Data points above the red line can be mainly explained by random flights or bees trying to escape and colliding with the transparent roof of the arena. (Right) Histogram of the flight probability along the z-axis of the arena. The data show that bees were flying close to the floor for most of the time, with 75% of the data laying below 17.13 cm. [file Image_1.TIFF]

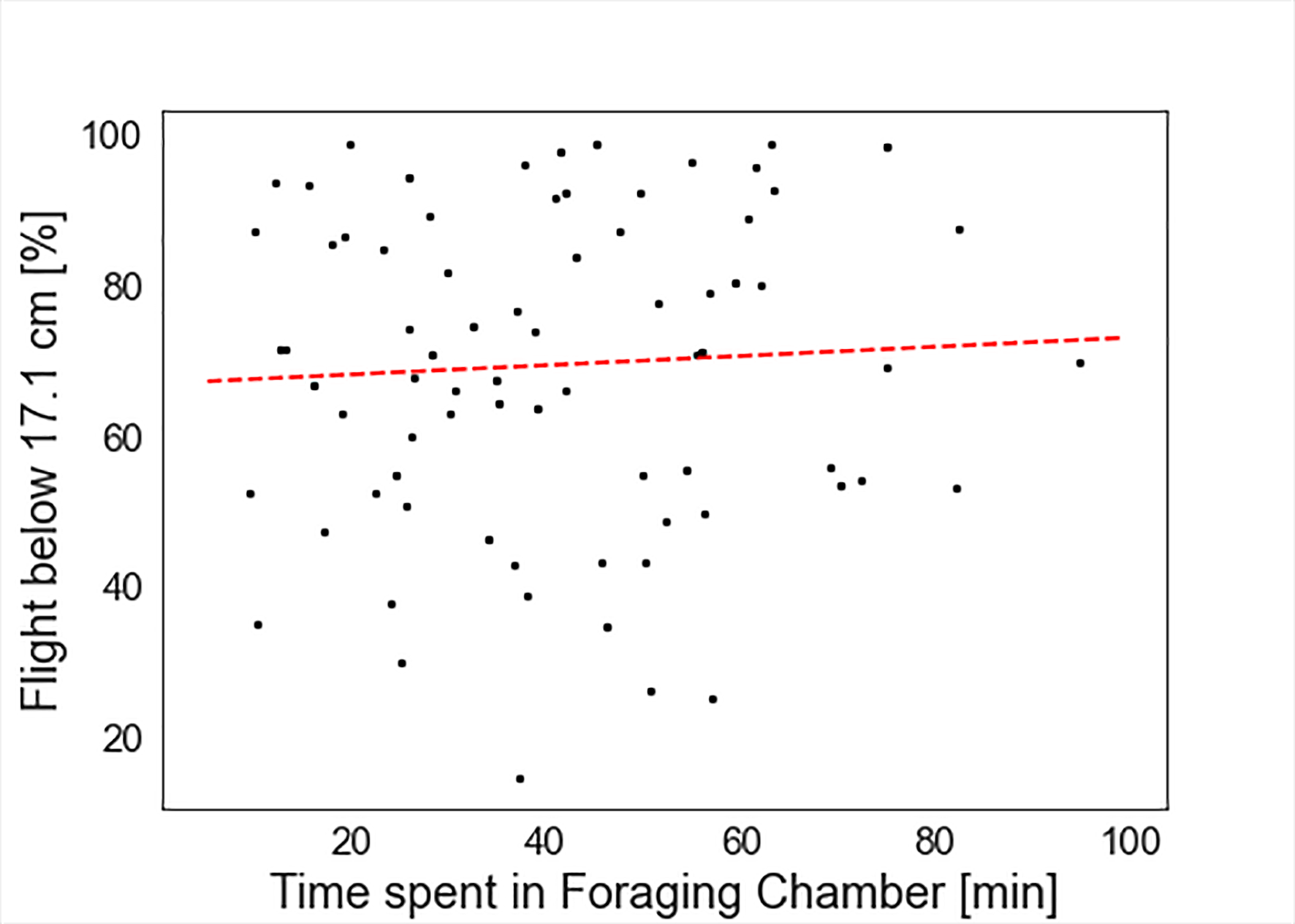

Supplement: Supplementary Figure 2 — Effect of the time the bees were blocked before their return flight on the duration of searching. The time spent in the foraging chamber has no significant effect on the search behavior, i.e., the time of the recording that was spent below 17.13 cm (linear regression; slope: 0.06, p > 0.1). [file Image_2.TIFF]
